# Discrepancy in CCL2 and CCR2 expression in white versus grey matter hippocampal lesions of Multiple Sclerosis patients

**DOI:** 10.1186/s40478-014-0098-6

**Published:** 2014-08-23

**Authors:** Marloes Prins, Ranjan Dutta, Bart Baselmans, John J P Brevé, John G J M Bol, Sadie A Deckard, Paul van der Valk, Sandra Amor, Bruce D Trapp, Helga E de Vries, Benjamin Drukarch, Anne-Marie van Dam

**Affiliations:** Department of Anatomy and Neurosciences, VU University Medical Center, Neuroscience Campus Amsterdam, Van der Boechorststraat 7, 1081 Amsterdam, BT The Netherlands; Department of Neurosciences, Cleveland Clinic, Lerner Research Institute, VU University Medical Center, Cleveland, OH USA; Department of Pathology, VU University Medical Center, Amsterdam, The Netherlands; Neuroimmunology Unit, Blizard Institute of Cell and Molecular Science, Barts and The London, School of Medicine and Dentistry, VU University Medical Center, London, UK; Department of Molecular Cell Biology & Immunology, VU University Medical Center, Amsterdam, The Netherlands

**Keywords:** Multiple sclerosis, Hippocampus, Monocyte chemotactic protein-1, Microglia, Astrocyte, Proliferation

## Abstract

A remarkable pathological difference between grey matter lesions (GML) and white matter lesions (WML) in Multiple Sclerosis (MS) patients is the paucity of infiltrating leukocytes in GML. To better understand these pathological differences, we hypothesize that the chemokine monocyte chemotactic protein-1 (MCP-1 or CCL2), of importance for leukocyte migration, and its receptor CCR2 are more abundantly expressed in WML than in GML of MS patients. To this end, we analyzed CCL2 and CCR2 expression in the hippocampus, comprising WML and GML, of post-mortem MS patients, and of control subjects.

CCL2 and CCR2 mRNA were significantly increased in demyelinated MS hippocampus. Semi-quantification of CCL2 and CCR2 immunoreactivity showed that CCL2 is present in astrocytes only in active WML. CCR2 is upregulated in monocytes/macrophages or amoeboid microglia in active WML, and in ramified microglia in active GML, although to a lesser extent. As a follow-up, we observed a significantly increased CCL2 production by WM-, but not GM-derived astrocytes upon stimulation with bz-ATP *in vitro*. Finally, upon CCL2 stimulation, GM-derived microglia significantly increased their proliferation rate.

We conclude that within hippocampal lesions, CCL2 expression is mainly restricted to WML, whereas the receptor CCR2 is upregulated in both WML and GML. The relative absence of CCL2 in GML may explain the lack of infiltrating immune cells in this type of lesions. We propose that the divergent expression of CCL2 and CCR2 in WML and GML explains or contributes to the differences in WML and GML formation in MS.

## Introduction

Multiple sclerosis (MS) is a chronic neuroinflammatory and degenerative disease affecting mostly young adults in the prime of their life. Clinical features are extremely varied and include cognitive deficits, e.g. memory impairment [1]. Pathologically, MS is characterized by areas of focal demyelination which are spread throughout the entire central nervous system [1]. These demyelinated lesions can be found in several white matter (WM) as well as grey matter (GM) areas, ranging from subcortical and subventricular WM, and tracts within the spinal cord [2-4] and of the brainstem [2] to GM structures such as the thalamus [5,6], hypothalamus [7], hippocampus [8,9], cerebellum [5,10] and cortex [5,6,11].

White matter lesions (WML) are characterized by the infiltration of large numbers of leukocytes [12-14], which are considered to play a crucial role in the formation of WML [15-17]. Although the presence of these cells has been described in GM lesions (GML) biopsy material of MS patients [18,19], a range of studies using post-mortem MS material demonstrated the relative absence of infiltrated immune cells in GML [11,20-22]. This difference in immune cell infiltration between post-mortem WML and GML is best illustrated by leukocortical lesions which encompass both WM and GM (type I lesions), with more T-cells, monocytes/macrophages and/or activated microglia present in the WM than GM part of the lesions [20].

Various hypotheses have been put forward to explain the apparent pathological differences between WML and GML, including a putative role for a divergent expression of chemo-attractant molecules important for the influx of immune cells into the brain. One such molecule is the chemokine monocyte-chemotactic protein-1 (MCP-1, also known as CCL2), which is reported to contribute to the pathogenesis of MS [23,24], through its involvement in the migration of leukocytes into the CNS [25-27]. In addition, CCL2 has been shown to induce migration [28,29], proliferation [30] and activation [30,31] of microglia *in vitro.* CCL2 mediates its effects by binding to and activating the chemokine receptor CCR2. CCL2-CCR2 interaction is essential to evoke the clinical and histopathological characteristics of experimental autoimmune encephalomyelitis (EAE), an animal model of MS, by regulating the infiltration of immune cells [26] and activation of microglial cells [32]. During EAE, a higher number of infiltrated immune cells correlated with increased disease severity [33] and blockade of CCL2-CCR2 signaling ameliorated progression of EAE together with a decrease in the number of infiltrating immune cells [34]. In post-mortem human MS tissue, CCL2 expressing astrocytes have been described to be present in WML [35-37] which may then contribute to the attraction of immune cells into WM leading to WML formation.

Based on the described histopathological differences between WML and GML, we hypothesize that CCL2 and its receptor CCR2 are more abundantly expressed in WML than in GML of MS patients. To this end, we studied post-mortem human hippocampus, a brain region known to be affected during MS, and to contain WML, GML, and mixed WML and GML [8,9]. We analyzed CCL2 and CCR2 expression in the hippocampus of MS patients and control subjects using semi-quantitative qPCR analysis and immunohistochemistry. An *in vitro* approach was used to study bz-ATP-induced CCL2 production by astrocytes derived from WM versus GM rat brain. Finally, we determined *in vitro* whether the CCR2 present in GM-derived microglia is functionally active, i.e. involved in CCL2 induced proliferation of microglial cells.

## Materials and methods

### Human subjects

*For semi-quantitative qPCR analysis*, post-mortem hippocampal tissue was collected as part of the “tissue procurement program” approved by the Cleveland clinic Institutional Review Board. Hippocampal fresh frozen tissue of 10 MS patients (age range: 40-73 years) and 5 control subjects (age range: 52-77 years) was included in this study and pathologically characterized previously [38]. Clinicopathological data of the MS patients and non-neurological controls used for qPCR are provided in Table 1.

**Table 1:** **Clinicopathological data MS patients and control subjects (qPCR study)**

**Case**	**Gender**	**Age**	**PMD (h)**	**DD**	**MS Type**	**Demyelination**	**COD**
**MS**							
1	F	58	7.3	13	RR	no	Abdominal Adenocarcinoma
2	F	54	5.9	37	SP	no	Unknown Infection
3	M	63	4.9	36	PP	no	Sepsis
4	F	54	7.1	5	SP	no	Drug Overdose
5	F	51	7.0	15	PP	no	Attempted Suicide (asphyxiation)
6	F	73	7.0	46	PP	yes	Dehydration/Gastroenteritis
7	F	46	5.6	14	SP	yes	Unknown
8	M	52	6.7	30	SP	yes	Pulmonary Infections
9	F	40	5.4	13	SP	yes	Pneumonia
10	F	66	13.0	35	SP	yes	Unknown
**Control**							
1	M	65	8.5				Cardiac arrest
2	F	53	36.0				Cardiac thrombosis
3	F	52	12.0				Polymicrobial sepsis
4	M	53	12.0				Myocardial infarct
5	M	77	12.0				Mesenteric bleeding

*PMD* = Postmortem delay; *DD* = Disease duration; *COD* = Cause of death; *SP* = Secondary progressive; *PP* = Primary progressive.

*For immunohistochemistry*, post-mortem human hippocampal tissue was obtained from the Netherlands Brain Bank (NBB, Amsterdam, The Netherlands) or from the Department of Pathology (VU University Medical Center in Amsterdam, The Netherlands). Formalin-fixed, paraffin-embedded hippocampal tissue sections were included from 18 clinically diagnosed and neuropathologically verified MS patients (age range: 43-77 years) and 9 control subjects (age range: 50-92 years) without neurological or psychiatric disease. Clinicopathological data of the MS patients and non-neurological controls used for immunohistochemistry are provided in Table 2.

**Table 2:** **Clinicopathological data MS patients and control subjects (immunohistochemistochemical study)**

**Case**	**Gender**	**Age**	**PMD (h)**	**DD**	**MS Type**	**Demyelination (lesion type)**	**COD**
**MS**							
11	M	43	8:30	17	SP	yes (active)	Pneumonia
12	F	66	6:00	23	SP	yes (active)	Unknown
13	F	60	10:40	7	PP	yes (active)	Euthanasia
14	M	54	8:15	12	PP	yes (active)	Euthanasia
15	F	50	7:35	17	SP	yes (active)	Euthanasia
16	F	62	6:45	25	Unknown	yes (inactive)	Unknown
17	M	63	7:05	28	SP	yes (inactive)	Cardiac arrest after rupture of abdominal aorta
18	M	47	7:15	7	SP	yes (inactive)	Urosepsis with organ failure
19	M	49	8:00	26	PP	yes (inactive)	Pneumonia by MS
20	M	66	7:30	26	PP	yes (inactive)	Ileus
21	M	61	9:15	31	SP	yes (inactive)	Euthanasia
22	F	57	8:40	27	Unknown	yes (inactive)	Respiratory insufficiency by (uro)sepsis
23	F	77	5:43	6	Unknown	no	Respiratory insufficiency with aspiration pneumonia
24	M	71	7:00	26	PP	no	Pneumonia by aspiration
25	F	76	9:45	20	PP	no	Unknown
26	M	50	9:30	24	PP	no	Unknown
27	M	51	11:00	20	Unknown	no	Unknown
28	M	56	9:50	14	PP	no	Cachexia and exhaustion by end stage MS
**Control**							
6	M	84	7:05				Exacerbation of COPD
7	M	56	9:15				Myocardial infarction
8	F	62	7:55				Euthanasia
9	F	92	7:00				Acute death, probably pulmonary embolism
10	F	50	4:10				Metastasized large cell bronchocarcinoma
11	F	62	7:20				Metastases
12	M	82	5:10				Pneumonia/cardiovascular accidents
13	M	78	Unknown				Cardiac arrest after rupture of abdominal aorta
14	F	84	6:55				Myelodysplasia

*PMD* = Postmortem delay; *DD* = Disease duration; *COD* = Cause of death; *SP* = Secondary progressive; *PP* = Primary progressive.

### qPCR analysis on human hippocampus material

Fresh hippocampal tissue was homogenized in Qiazol and total RNA was isolated as described by the manufacturer using the RNeasy Microarray tissue kit (Qiagen Inc, Valencia, USA). RNA concentration was determined spectrophotometrically at 260 nm using the NanoDrop 2000 spectrophotometer (Thermo Fisher Scientific, Waltham, USA) and purity of the RNA samples was determined by measuring the absorbance ratio at 260/230 nm (samples were excluded when ratio was outside the 2.00-2.20 range) and 260/280 nm (samples were excluded when ratio was outside the 1.90.-2.10 range). When RNA quality was approved, 1 μg of RNA was reverse transcribed into cDNA and the PCR reaction was carried out using RT^2^ First Strand kit (Qiagen Inc, Valencia, USA) according to the manufacturer’s instructions. For the qPCR reaction, qPCR assays (CCL2:PPH00192E; CCR2:PPH00612F) were purchased (Qiagen Inc, Valencia, USA). All reactions were normalized to the reference gene glyceraldehyde-3-phosphate dehydrogenase (GAPDH) using the qPCR Assay (PPH00150E, Qiagen Inc.), which have been found to produce reproducible results using RT-PCR analysis previously performed using control and MS hippocampus tissues [38,39].

Amplification of cDNA was performed in MicroAmp Optical 96-well Reaction Plates (Applied Biosystems Inc, NY, USA) and the analysis was done using an ABI Biosystems 7300 RT-PCR System (Applied Biosystems Inc, NY, USA). The 25 μl reaction mixture was composed of RT^2^ SYBR Green Rox Master Mix (Qiagen Inc.), 1 μl cDNA and 1 μl of the forward and reverse primers each. As a negative control, no template (no cDNA) reactions were performed to exclude DNA contamination, and to confirm the absence of primer-dimer artifacts from amplification plots. The reaction conditions were an initial 10 min at 95°C, followed by 40 cycles of 15 sec at 95°C and 1 min at 60°C each. All samples were run in triplicate and mRNA levels were normalized to the levels of the reference gene GAPDH using previously published methods [38,39].

### Immunohistochemical detection of MBP, MHC-II, CCL2 and CCR2

After rapid autopsy (mean postmortem delay: 7.5 h) hippocampal tissue samples were fixed in 10% formalin for 30 days and embedded in paraffin. Of 32 paraffin-embedded tissue blocks, 5-μm sections were cut and mounted on positively charged glass slides (Menzel-Glaser SuperFrost plus, Braunschweig, Germany), and dried overnight at 37°C. Upon use, sections were heated in an incubator for 30 min at 56°C, before they were deparaffinized in xylene, and rehydrated through a series of 100%, 96%, and 70% ethanol and distilled water. For subsequent antigen retrieval, sections were rinsed in 0.01 M citrate buffer (pH 6.0) or in 10 mM Tris buffer (pH 9.0) containing 1 mM EDTA (Tris-EDTA) and subsequently heated in a steamer for 30 min at 90-99°C in the same buffers. After antigen retrieval, the sections were allowed to regain room temperature (RT), rinsed in Tris-buffered saline (TBS), and incubated for 20 min in TBS containing 0.3% H_2_O_2_ and 0.1% sodiumazide. Non-specific binding sites were blocked with 5% non-fat dried milk (Campina) in TBS containing 0.5% Triton (TBS-T; blocking solution) for 30 min at RT. Subsequently, sections were incubated overnight at 4°C with the following primary antibodies: myelin basic protein (MBP), MHC-II (LN3), CCL2 or CCR2 (see Table 3 for details on primary antibodies), diluted in blocking solution. The next day, sections were washed in TBS and incubated for 2 h at RT in corresponding biotinylated IgG’s (1:400, Jackson Immunoresearch, Westgrove, PA, USA; see Table 3 for details on secondary antibodies), followed by HRP-labeled avidin-biotin complex (ABC complex 1:400; Vector Laboratories, Burlingame, CA, USA) for 1 h at RT. To detect CCL2 immunoreactivity, an amplification step was included at this stage, and thus sections were subsequently incubated in biotinylated tyramide (1:800, kindly provided by Dr. I. Huitinga, Netherlands Institute for Neuroscience, Amsterdam) and 0.01% peroxide in TBS-T for 30 min, followed by another incubation with ABC complex (1:800) in TBS-T for 1 h. Detection of all antigens was visualized using 3,3-diaminobenzidine (DAB, Sigma, St. Louis, MO, USA) as a chromogen and counterstaining was performed with hematoxylin. After dehydration in graded ethanol solutions, the sections were cleared in xylene and coverslipped in Entellan (Merck, Darmstadt, Germany). Negative controls were performed by omitting the primary antibody resulting in no immunohistochemical signal (data not shown).

**Table 3:** **Primary and secondary antibodies used for single labeling**

**Antigen**	**Antigen retrieval**	**Species**	**Final dilution**	**Source primary antibody**	**Secondary antibody**	**Source secondary antibody**
Human MBP	Tris-EDTA	Mouse	1:100	Boehringer, 1118099	Biotinylated donkey-anti-mouse IgG	Jackson, 715-065-151
Human MHC-II	Citrate	Mouse	1:100	Gift, Clone LN3	Biotinylated donkey-anti-mouse IgG	Jackson, 715-065-151
Human CCL2	Citrate	Mouse	1:200	R&D Systems, MAB2791	Biotinylated goat-anti-mouse IgG	Jackson, 115-065-146
Human CCR2	Citrate	Rabbit	1:1000	Abcam, ab32144	Biotinylated goat-anti-rabbit IgG	Jackson, 111-065-144

### Immunofluorescent double labeling

Based on the morphological appearance of CCL2 and CCR2 positive cells, double-labeling of astrocytes and CCL2 or of microglia and CCR2 was performed. To that end, antibodies for astrocytes, i.e. glial fibrillary acidic protein (GFAP), and for CCL2 or antibodies for microglia, i.e. ionized calcium-binding adapter molecule 1 (Iba-1) and for CCR2 were used. In addition, a double labeling of CCR2 and the cell proliferation marker BM28 [40] was performed.

Sections were co-incubated (Iba-1/CCR2 and Iba-1/BM28) with the appropriate primary antibodies. To prevent steric hindrance of the CCL2 antibody and GFAP antibody, sections were sequentially incubated (GFAP/CCL2) with the appropriate primary antibodies (see Table 3 for details on primary antibodies used).

Sections were deparaffinized and antigen retrieval was performed with citrate or Tris-EDTA buffer as described above. Non-specific binding sites were blocked with 3% bovine serum almunin (BSA) (Sigma) in TBS-T for 30 min at RT. All antibodies were diluted in 3% BSA in TBS-T. After a 24 h incubation at 4°C, the sections were washed and subsequently incubated for 90 min at RT with appropriate Alexa Fluor 488 or Alexa Fluor 594 labeled IgG’s (1:400; Molecular Probes) or with streptavidin-labeled Alexa Fluor 488 (1:400, Molecular Probes) when the secondary antibodies were biotinylated (see Table 4 for detailed information on secondary antibodies and conjugates). After washing, the sections were coverslipped with Vectashield (Vector Laboratories, Burlingame, CA, USA). Sections were examined using a confocal laser scanning microscope (Leica TSC-SP2-AOBS; Leica Microsystem, Wetzlar, Germany).

**Table 4:** **Primary and secondary antibodies used for double labeling**

**Antigen**	**Antigen retrieval**	**Species**	**Final dilution**	**Source primary antibody**	**Secondary antibody**	**Source secondary antibody**	**Conjugate**	**Source conjugate**
Iba-1	Tris-EDTA	Goat	1:300	Abcam, ab5076	Alexa Fluor 488 coupled donkey anti goat IgG	Molecular Probes, A11055		
GFAP	Citrate	Rabbit	1:4000	DAKO, Z0334	Alexa Fluor 594 coupled donkey anti rabbit IgG	Molecular Probes, A21207		
CCL2	Citrate	Mouse	1:200	R&D Systems, MAB2791	Biotinylated goat anti mouse IgG	Jackson, 115-065-146	Alexa Fluor 488 coupled streptavidin	Molecular Probes, S11223
CCR2	Tris-EDTA	Rabbit	1:1000	Abcam, ab32144	Alexa Fluor 594 coupled donkey anti rabbit IgG	Molecular Probes, A21207		
BM28	Tris-EDTA	Mouse	1:600	BD Transduction Laboratories, 610700	Biotinylated donkey anti mouse IgG	Jackson, 715-065-151	Alexa Fluor 488 coupled streptavidin	Molecular Probes, S11223

### Definition of lesion center and border

**Figure 1:**
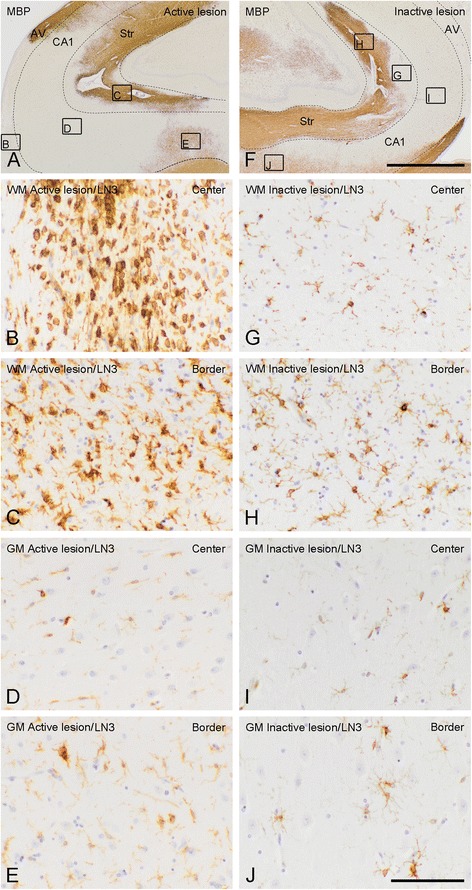
**Histopathological features of active and inactive hippocampal MS lesions.** MS lesions are recognized by the loss of MBP immunoreactivity in hippocampal WM and/or GM areas **(A,F)**. Active and inactive lesions were distinguished based on the presence or absence of MHC-II positive monocytes/macrophages, respectively. WM within the lesion centre **(B)** and at the border **(C)** of the active lesion presents with activated amoeboid MHC-II positive cells. The number of MHC-II positive cells in the lesion centre **(D)** and at the border of a GML **(E)** is far lower compared to WML. Moreover, LN3^+^ cells within the GM have a more ramified morphology. LN3^+^ cells within an inactive lesion are lower in number compared to active lesions and have a ramified morphology, both within the centre **(G)** and at the border **(H)** of WM, as well as in the centre **(I)** and at the border of **(J)** GM. Frames in A & F refer to hippocampal areas shown in B-E; G-J. The dashed lines indicate the border between WM (alveus and striatum) and GM (CA1). AV = alveus; Str = Striatum. Scale bar **(A, F)** = 500 μm. Scale bar **(B-E; G-J)** = 60 μm.

Lesions were identified by the loss of myelin basic protein (MBP) immunoreactivity. The border of a lesion is represented by the edge of demyelinated and myelinated WM or GM. The center of a lesion is the demyelinating or demyelinated area between the borders of a WML or a GML (Figure 1A, F). A distinction was made between demyelinated WM and GM regions within the hippocampus, in which the WM regions comprise the *stratum radiatum* and the alveus and GM regions comprise the *cornu ammonis* (CA) 1, CA2, CA3 and CA4. The activity status was determined by the presence or absence of MHC-II positive monocytes/macrophages or microglia in the border or center of a lesion. Active/chronic active and inactive WML were defined as described before [41,42], in which active and chronic active lesions show amoeboid monocytes/macrophages in the center (Figure 1B) or at the border (Figure 1C) of the lesions, respectively, while inactive lesions are almost devoid of activated immune cells, but do show ramified microglia (Figure 1G, H) [41-43]. The activity status of GML, which are virtually devoid of immune cells, was determined by the presence or absence of MHC-II positive amoeboid macrophages present within neighboring demyelinated WM which was part of the same lesion (combined WML and GML) and the presence of MHC-II positive microglia in the GML. Although not officially identified as such, we called these active GML (Figure 1D, E). When hardly any MHC-II positive microglia were present, we called these inactive GML (Figure 1I, J). Thus, to be able to carefully localize the CCL2 and CCR2 expressing cells, we discriminated between the center and the borders of WML and GML as well as between active/chronic active and inactive lesions.

### Semi-quantitative analysis of CCL2- and CCR2-positive cells

Semi-quantification of CCL2- and CCR2-positive cell numbers was performed by unbiased manually counting the number of positive cells with a clearly visible cell nucleus within the region of interest (ROI). In MS lesions, ROIs were positioned at the white matter border, grey matter border, white matter lesion center and grey matter lesion center. The CA4 region was excluded from analysis since this region often showed nonspecific astrocytic staining regardless of the presence of lesions. In control subjects and MS patients without hippocampal lesion, ROIs were placed in hippocampal WM and GM. In each ROI, cells were counted in 2 fields, each measuring 0.1-0.4 mm^2^. Results were expressed as number of cells (mean and standard error of the mean) per mm^2^. All pictures were acquired using an Olympus-VANOX-T microscope (Tokyo, Japan) at 10-fold magnification. Cell counting and area determination were performed using Cell^F Olympus Soft Imaging Software (Tokyo, Japan).

### Primary culture of astrocytes and microglia

Primary astrocytes and microglia were isolated from 1 day old Wistar rats (Harlan CPB, Zeist, The Netherlands) as described previously [44] and which was approved by the Animal Experiment Committee of the VU University Medical Center, Amsterdam, The Netherlands (approval ID: FGA 11-03). Mixed glial cells were isolated and cultured from pons and cerebral cortices to obtain mainly WM and GM primary astrocytes, respectively. These brain regions were cleared from adhering meninges and blood vessels, and mechanically dissociated in Dulbecco’s modified Eagle’s medium (DMEM)-F10 (Gibco, Life Technologies, Breda, The Netherlands), supplemented with 10% v/v heat-inactivated fetal calf serum (FCS) (Gibco), 2 mM L-glutamine (Sigma-Aldrich), 50 Units/ml penicillin (Sigma-Aldrich) and 50 μg/ml streptomycin (Gibco). Cells were plated in poly-L-lysine (15 μg/ml (2 μg/cm^2^); Sigma-Aldrich) coated T75 culture flasks (Nunc, Hamstrop, Denmark) and incubated at 37°C in humidified air containing 5% CO2. The medium was changed at day 1, day 6 and day 8 after seeding. After 10 days in culture, microglia and astrocytes were separated by shaking the flasks at 37°C at 260 rpm for 16 h. Cortical microglia were directly plated into poly-L-lysine coated 8-well chamber-slides (Lab-Tek). Astrocytes were further purified by treatment with 5 mM leucine methyl ester (LME, Sigma- Aldrich) in serum free medium, for 24 h at 37°C. Subsequently, cortical and pons-derived astrocytes were plated in poly-L-lysine coated 6-well plates (Thermo Scientific) containing fresh medium with serum. Purity of astrocyte cultures was between 80-90% as determined by immunofluorescent staining using anti-GFAP antibody (1:6000; DAKO; Z0334). Purity of primary microglia cultures was between 90-94% as determined by immunofluorescent staining using anti-Iba1 antibody (1:1000;WAKO Chemicals USA; 019-19741).

### Bz-ATP-induced CCL2 expression in white and grey matter derived astrocytes

To determine the level of CCL2 expression by white and grey matter derived astrocytes, 0.5 x 10^6^ cells were plated onto poly-L-lysine coated wells for 24 h at 37°C. Astrocytes were cultured in serum-free medium alone (control) or in the presence of Benzoyl-benzoyl adenosine 5’-triphosphate (bz-ATP) (500 μM) for 2 to 6 h (n = 3 independent experiments). Bz-ATP is a potent analogue of ATP and was chosen as a stimulus because ATP release is a physiological response upon axonal damage [45], such as observed in WML and GML in MS [20,42]. Subsequently, ATP (and bz-ATP) acts on purinergic receptors, e.g. P2X7, present on astrocytes [46] resulting in various responses, including CCL2 production [47]. After incubation with bz-ATP, astrocytes were homogenized in Trizol reagent (Life Technologies, Carlsbad, USA) and total RNA was isolated as described by the manufacturer. RNA concentration was determined spectrophotometrically at 260 nm using the NanoDrop ND-1000 spectrophotometer (Thermo Fisher scientific, Waltham, USA) and purity was determined by measuring the absorbance ratio at 260/280 nm, and was approved when values were between 1.9 and 2.1. When quality criteria were met, 1 μg of RNA was reverse transcribed into cDNA using the High Capacity cDNA Reverse Transcription Kit (Invitrogen) according to the manufacturer’s instructions, but using oligo-d(T)_16_ primers (Applied Biosystems). For the qPCR reaction, primers for CCL2 (GenBank accession number: NM_031530.1) and GAPDH (GenBank accession number: NM_017008.3) were designed and purchased from Eurogentec (Seraing, Belgium).

GAPDH was identified as the most stable reference gene compared to four other reference genes, i.e. hypoxanthine phosphoribosyltransferase 1 (HPRT1), phosphoglycerate kinase 1 (PGK1), peptidylprolyl isomerase A (cyclophilin or PPIA) and tyrosine 3-monooxygenase/tryptophan 5- monooxygenase activation protein, zeta polypeptide (YWHAZ).

Details of the primer sequences are as follows:

- CCL2 forward primer: 5′-ACGTGCTGTCTCAGCCAGATG-3′
- CCL2 reverse primer: 5′-GACTCATTGGGATCATCTTGCC-3′
- GAPDH forward primer: 5′-GAACATCATCCCTGCATCCA-3′
- GAPDH reverse primer: 5′-GCCAGTGAGCTTCCCGTTCA-3′

Amplification of cDNA was performed in MicroAmp Optical 96-well Reaction Plates (Applied Biosystems) and the analysis carried out using a StepOnePlus Real-Time-PCR System (Applied Biosystems). The 20 μl reaction mixture was composed of Power SYBR Green PCR Master Mix (Applied Biosystems), 12.5 ng cDNA and 4 pM of the forward and reverse primers each. As a negative control, no template (no cDNA) reactions were performed. The reaction conditions were an initial 2 min at 50°C, followed by 10 min at 95°C and 40 cycles of 15 sec at 95°C and 1 min at 60°C. The mRNA expression levels were quantified relatively to the level of the reference gene glyceraldehyde-3-phosphate-dehydrogenase (GAPDH) using the following calculation: 2^-(Cq of target mRNA - Cq of GAPDH)^ × 100%.

### Bromodeoxyuridine labelling assay

In contrast to hippocampal WM, hardly any CCL2 is present in hippocampal GM as observed in the present study (see Results section). We thus questioned whether the CCR2 receptor on GM-derived microglia is activated by CCL2 and results in a functional response. To this end, the effect of CCL2 treatment on GM microglia proliferation was studied. Cortical primary rat microglia were plated on poly-L-lysine coated chamberslides (2 × 10^4^ cells/chamber) and allowed to adhere for 2 h. Then, medium was refreshed and 10 nM of INCB3344 (MedChem Express, Princeton, USA), a specific CCR2 antagonist [48,49] or vehicle (medium) together with 20 μM bromodeoxyuridine (BrdU; Sigma) were added. After 30 min, 50 ng/ml of recombinant rat MCP-1 (CCL2; Peprotech) was added (n = 3 independent experiments). After 48 h, the cells were washed and subsequently fixed with 4% PFA for 20 min, and again washed three times with 1% Triton X-100 (Sigma-Aldrich) in PBS. Then, the fixed cells were incubated with 5 units/100 μl DNAse (Promega, H6101) for 45 min at 37°C. Subsequently, the fixed cells were blocked with 5% normal goat serum in PBS/1% Triton X-100 for 1 h. To identify BrdU incorporation, microglia were incubated with rat anti-BrdU (1:200; Abcam; AB6326) in PBS/1% Triton X-100 for 24 h at RT, washed with PBS/1% Triton X-100 followed by incubation with Alexa Fluor-488 labeled goat anti-rat IgG’s (1:200; Molecular Probes) for 2 h at RT. Cells were washed three times with PBS/1% Triton X-100 and once with PBS, treated for 5 min with 5 μg/ml propidiumiodide (PI) and again washed two times with PBS. Then, the slides were embedded in Vectashield (Vector Laboratories). Immunofluorescent staining was visualized using an Olympus VANOX-T microscope (Tokyo, Japan). In five random 20 × fields per well, the percentage of proliferative cells was calculated as follows: (number of BrdU positive cells/ number of PI positive nuclei) × 100%.

### Statistics

Statistical analyses were carried out with SPSS package version 20.0 (Statistical Product and Service Solutions, Chicago, IL, USA). Data of multiple tissue blocks from the same patient were averaged to finally represent each case with 1 value per ROI. Normal distribution of the data was tested using the Shapiro-Wilk procedure. When data were normally distributed, differences between control subjects, MS patients without hippocampal lesions, MS patients with active/chronic active hippocampal lesions and MS patients with inactive hippocampal lesions were compared using one-way ANOVA, followed by Tukey’s post hoc analysis. When data were not normally distributed, a Kruskal-Wallis test was performed followed by a Mann-Whitney U post-hoc analysis.

Differences in CCL2 and CCR2 immunoreactivity within subject WM vs GM were analyzed for controls, MS patients without and with hippocampal demyelination using the Wilcoxon Signed Rank Test. The Wilcoxon Signed Rank Test was also used for analyzing the difference between the number of CCL2 and CCR2 expressing cells within subject lesion center versus lesion border of MS patients with hippocampal demyelination. For evaluating the response of WM and GM astrocytes to bz-ATP, a 2-way ANOVA was performed followed by a Student’s t-test (Bonferroni adjusted). Proliferation of microglia was analyzed using a one-way ANOVA, followed by LSD post hoc analysis. P-values < 0.05 were considered significant.

## Results

### Lesion characterization

Areas of hippocampal demyelination were identified by loss of MBP immunoreactivity (Figure 1A, F). No demyelination was observed in the sections of non-neurological control subjects (not shown). Of the 18 MS patients, 6 did not show demyelination in the hippocampal tissue block obtained. The other 12 MS patients showed demyelinated areas in hippocampal tissue blocks studied, which were subsequently examined for the presence of MHC-II positive cells to determine the lesion activity. Of these, 5 were classified as having active/chronic active lesions. Together, these 5 patients had 11 lesions, of which 6 mixed WM/GM lesions, 1 purely GM lesion, and 4 purely WM lesions. Seven MS patients were classified as having inactive lesions. In total, this group comprised 22 lesions, of which 12 mixed WM/GM lesions, 6 purely GM lesions, and 4 purely WM lesions. In agreement with previous studies [9,50], only a minimal number of CD3 positive T-cells was observed in active WM lesions, whereas these cells were absent in active GM lesions (data not shown).

### *CCL2 and CCR2 mRNA* in human hippocampus

**Figure 2:**
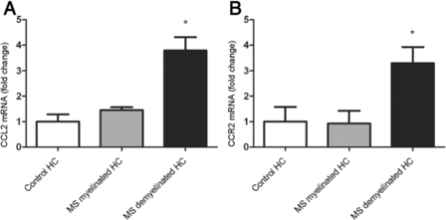
**Semi-quantitative RT-PCR analysis of CCL2 and CCR2 mRNA.** qPCR of CCL2 **(A)** and CCR2 **(B)** mRNA in the hippocampus of control subjects, MS patients without hippocampal lesions and MS patients with hippocampal lesions. Data represent mean ± S.E.M. (n = 5) and are expressed relative to GAPDH.

Levels of CCL2 and CCR2 mRNA within the hippocampus of MS patients and control subjects were determined by qPCR. While CCL2 and CCR2 mRNA in myelinated MS hippocampi (n = 5) was not altered compared to control hippocampi (n = 5), a ~4 fold increase in CCL2 mRNA (ANOVA; F = 18.07; p < 0.001) and a ~3 fold increase in CCR2 mRNA (ANOVA, F = 5.63; p = 0.02) was measured in demyelinated hippocampi (n = 5) compared to myelinated MS hippocampi (*p* < 0.001; p = 0.03, respectively) and compared to control hippocampi (*p* < 0.001; p = 0.04, respectively) (Figure 2A, B).

### Semi-quantitative analysis of CCL2 and CCR2 positive cells

For semi-quantitative analysis CCL2 and CCR2 positive cells were counted in several ROIs (see Material and methods). When more tissue blocks of one patient were available, scores for the same ROI in different tissue blocks were averaged. For each group, i.e. control subjects, MS patients without hippocampal lesions, MS patients with active/chronic active hippocampal lesions and MS patients with inactive hippocampal lesions, the mean and standard deviation of CCL2 and CCR2 positive cell numbers per ROI per case are summarized in Table 5.

**Table 5:** **Mean CCL2** ^**+**^ **and CCR2** ^**+**^ **cell numbers**

**Group**	**Location**	**Number of CCL2** ^**+**^ **cells (mean ± SD) per mm** ^**2**^		**Number of CCR2** ^**+**^ **cells (mean ± SD) per mm** ^**2**^	
		**WM**	**GM**	**WM**	**GM**
Control (n = 9)	Na	6.6 ± 9.1	1.0 ± 2.9	5.0 ± 7.5	1.9 ± 1.9
NDH (n = 6)	Na	8.4 ± 11.6	0 ± 0	9.1 ± 8.5	1.1 ± 2.6
Inactive lesions (n = 7)	Center	8.2 ± 9.7	0.7 ± 1.9	20.1 ± 25.2	1.4 ± 2.4
	Border	16.1 ± 23.8	1.8 ± 3.3	58.0 ± 84.8	2.3 ± 3.9
Active lesions (n = 5)	Center	21.1 ± 20.2	0.1 ± 0.3	189.8 ± 152.6	21.8 ± 21.4
	Border	45.3 ± 21.7	0 ± 0	256.6 ± 90.2	63.3 ± 14.3

*Na* = Not applicable; *NDH* = Non-demyelinated hippocampi; *SD* = Standard deviation.

#### CCL2

**Figure 3:**
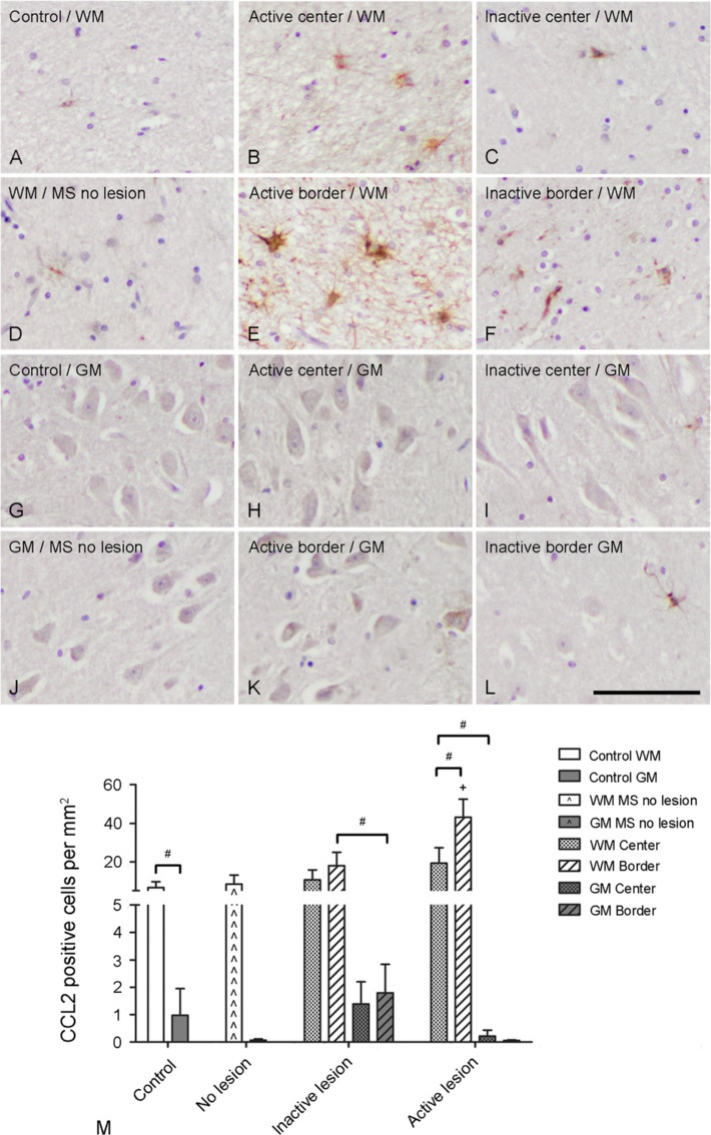
**Semi-quantitative analysis of CCL2 positive cell numbers.** CCL2 positive cells were only sporadically found within the hippocampal WM of control subjects **(A)** and MS patients without hippocampal lesions **(D)**. The number of CCL2 positive cells was slightly increased within the WM centre of active **(B)** and inactive **(C)** lesions and borders **(F)**. A significant increase in the number of CCL2 positive cells was found within the WM border of active lesions **(E)**. CCL2 positive cells were barely detected within the hippocampal GM of control subjects **(G)**, MS patients without hippocampal lesions **(J)**, the lesion centre **(H)** and border **(K)** of active lesions and only sporadically within the lesion centre **(I)** and border **(L)** of inactive lesions. Semi-quantification of CCL2 positive cells **(M)**. Scale bar **(A-L)** = 60 μm; + p < 0.05 versus cell number in WM of non-demyelinated hippocampi of MS patients and in hippocampal WM of control subjects; # p < 0.05.

In hippocampal WM of control subjects and in WM of myelinated hippocampi of MS patients a few CCL2 positive cells were found upon immunohistochemical analysis (Figure 3A, D). However, at the border of active demyelinated WML an increase in CCL2 positive cells was observed (45.3 ± 21.7 cells/mm^2^; Figure 3E), which was significantly different from the hippocampal WM of control subjects (6.6 ± 9.1 cells/mm^2^) and WM of myelinated hippocampi of MS patients (8.4 ± 11.6 cells/mm^2^) (Kruskall-Wallis, *p* = 0.031; Mann Whitney U, *p* = 0.004 and *p* = 0.017, respectively) (Figure 3 M). CCL2 positive cells were found within the center of active WML (21.1 ± 20.2 cells/mm^2^) (Figure 3B), albeit to a lesser extent, as well as in the center (Figure 3C) and at the border (Figure 3 F) of inactive WML. In the hippocampal GM areas only scarce CCL2 immunoreactivity was observed, irrespective of the absence or presence of GML (Figure 3G-L).

When we compared CCL2 immunoreactivity in WM and GM of control subjects, MS patients without hippocampal lesions and MS patients with active or inactive lesions, we found significantly more CCL2 positive cells in hippocampal WM (6.6 ± 9.1 cells/mm^2^) than GM (1.0 ± 2.9 cells/mm^2^) of control subjects (Wilcoxon Signed Rank test, *p* = 0.04). Moreover, in the center of active WML more CCL2 positive cells (21.1 ± 20.2 cells/mm^2^) were found than in the center of active GML (0.1 ± 0.3 cells/mm^2^) (Wilcoxon Signed Rank test, *p* = 0.04). CCL2 immunoreactive cells within the WM border of inactive lesions (16.1 ± 23.8 cells/mm^2^) were significantly outnumbering those found within the hippocampal GM border of inactive lesions (1.8 ± 3.3 cells/mm^2^) (Wilcoxon Signed Rank test, *p =* 0.04) (Figure 3M).

Finally, the number of CCL2 positive cells was significantly higher within the WM border compared to the center of active WM hippocampal lesions (Wilcoxon Signed Rank test, *p =* 0.04) (Figure 3M).

#### CCR2

**Figure 4:**
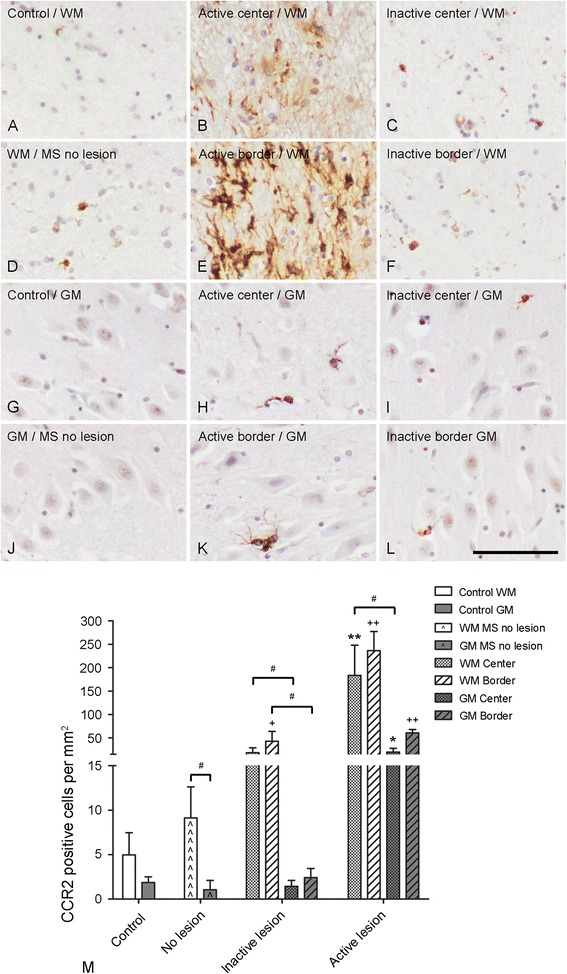
**CCR2 immunoreactivity.** Low numbers of CCR2 positive cells were observed within the hippocampal WM of control subjects **(A)**, MS patients without hippocampal lesions **(D)**, the lesion centre **(C)** and border **(F)** of inactive lesions. A significant increase in the number of CCR2 positive cells was found within the WM centre **(B)** and border **(E)** of active lesions. CCR2 positive cells were barely detected within the hippocampal GM of control subjects **(G)** and MS patients without hippocampal lesions **(J)**. The number of CCR2 positive cells was slightly increased within the WM centre of active **(H)** and inactive **(I)** lesions and borders **(L)**. A significant increase in the number of CCR2 positive cells was found within the GM border of active lesions **(K)**. Semi-quantification of CCR2 positive cells **(M)**. Scale bar **(A-L)** = 60 μm. + p < 0.01 compared to number in the WM of control subjects and the WM border of active lesions, ++ p < 0.01 compared to number in the GM of control subject and the GM border of inactive lesions and +++ p < 0.01 compared to number in the WM border of inactive lesions and WM of non-demyelinated hippocampi of MS patients and control subjects. * p < 0.05 compared to numbers in the GM center of inactive lesions and GM of MS patients without hippocampal lesions and control subjects, and ** p < 0.01 compared to numbers in the WM center of inactive lesions and WM of MS patients without hippocampal lesions and control subjects. ; # p <0.05.

CCR2 positive cells were found infrequently within the WM of control subjects and myelinated MS hippocampi (Figure 4A, D). However, within the center (Figure 4B) and at the border (Figure 4E) of active demyelinated WML, CCR2 positive cells were abundantly present. In inactive WML, CCR2 expression was still present but to a lesser extent within the center (Figure 4C) and at the border (Figure 4F) of the lesion. Semi-quantification indicated that inactive hippocampal WML presented with significantly more CCR2 positive cells within the border (58.0 ± 84.8 cells/mm^2^) compared to hippocampal WM of control subjects (5.0 ± 7.5 cells/mm^2^) (ANOVA, F = 19.77, *p* < 0.001; Tukey HSD, *p* = 0.008). Active hippocampal WML showed significantly more CCR2 immunopositive in the WM border (256.6 ± 90.2 cells/mm^2^) compared to WM in control hippocampi, and WM in myelinated MS hippocampi (9.1 ± 8.5 cells/mm^2^) and the WML and border of inactive hippocampal lesions (ANOVA, F = 19.77, *p* < 0.001; Tukey HSD, *p* < 0.005 for all comparisons). Similarly, the center of active WML (189.8 ± 152.6 cells/mm^2^) showed significantly more CCR2 positive cells than WM in control hippocampi, WM in myelinated MS hippocampi and the center of inactive WML (20.1 ± 25.2 cells/mm^2^) (Kruskal-Wallis, *p* = 0.003; Mann-Whitney U, *p* = 0.001, *p* = 0.004 and *p* = 0.003, respectively).

In contrast to the absence of CCR2 positive cells in hippocampal GM of control subjects and in GM of myelinated hippocampal tissue of MS patients (Figure 4G, J), CCR2 immunoreactive cells appeared within the center and at the border of active GML (Figure 4H, K). Less CCR2 positive cells were found in inactive GML (Figure 4I, L). After semi-quantification we measured a significant increase in CCR2 immunoreactivity within the hippocampal GML center (21.8 ± 21.4 cells/mm^2^) and border (63.3 ± 14.3 cells/mm^2^) of active lesions compared to hippocampal GM of control subjects (1.9 ± 1.9 cells/mm^2^), GM of myelinated hippocampi of MS patients (1.1 ± 2.6 cells/mm^2^) and GML center (1.4 ± 2.4 cells/mm^2^) and border (2.3 ± 3.9 cells/mm^2^) of inactive lesions (Kruskal-Wallis, *p* = 0.01; Mann-Whitney U, *p =* 0.01, *p* = 0.009 and *p* = 0.01, respectively; Kruskal-Wallis, p = 0.007; Mann-Whitney U, *p* < 0.003; *p* = 0.01 and *p* = 0.006, respectively) (Figure 4M).

When we compared CCR2 immunoreactivity in WM and GM of control subjects, MS patients without hippocampal lesions and MS patients with active or inactive lesions we found that the number of CCR2 positive cells was significantly higher in hippocampal WM compared to GM of MS patients without hippocampal lesions (Wilcoxon Signed Rank test, *p* = 0.03). In addition, CCR2 positive cells were significantly more numerous in the WML center compared to the GML center of active and inactive hippocampal lesions (Wilcoxon Signed Rank test, *p* = 0.04 and p = 0.05, respectively). Furthermore, the number of CCR2 positive cells was significantly higher within the WML border compared to GML border of hippocampal inactive lesions (Wilcoxon Signed Rank test, *p* = 0.02) (Figure 4M).

#### Cellular identification of CCL2 and CCR2 immunoreactivity

**Figure 5:**
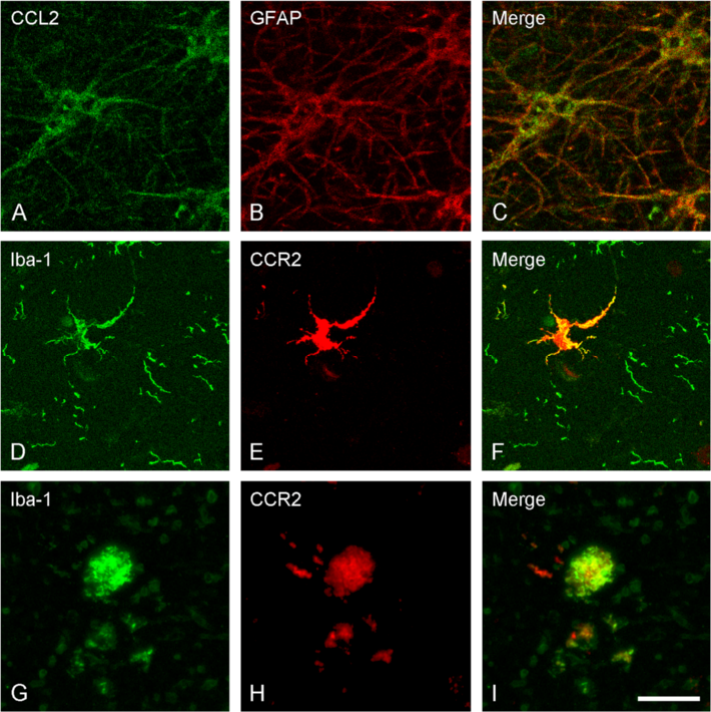
**Cellular localization of CCL2 positive and CCR2 positive cells.** Representative images of confocal laser scanning microscopy revealed colocalization of CCL2 with GFAP **(A-C)**, whereas CCR2 colocalized with ramified iba-1 positive cells in hippocampal GM **(D-F)**, and amoeboid iba-1 positive cells in hippocampal WM **(G-I)**. Scale bar **(A-F)** = 10 μm.

Based on the morphological appearance of CCL2 and CCR2 positive cells in hippocampal MS lesions, we performed double labeling studies on lesioned hippocampal material to determine whether CCL2 is expressed by astrocytes and CCR2 by microglia. The experiments revealed extensive co-localization of CCL2 with GFAP in all lesion types (Figure 5A-C), demonstrating that astrocytes indeed expressed CCL2. Additionally, CCR2 co-localized with iba-1 positive ramified microglia in WML and GML (Figure 5D-F), and with infiltrating monocytes/macrophages or amoeboid microglia in WML (Figure 5G-I).

### Primary culture of astrocytes and microglia

#### Bz-ATP-induced CCL2 mRNA expression by WM- and GM-derived astrocytes

**Figure 6:**
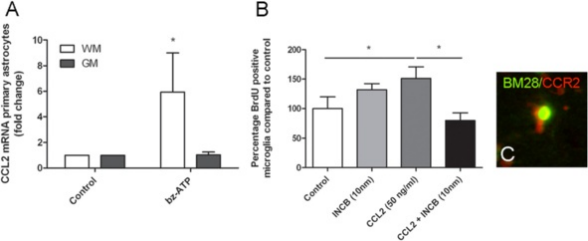
**CCL2 mRNA in astrocytes and CCL2 induced microglial proliferation** ***.*** Primary WM rat astrocytes produce significantly more CCL2 mRNA upon treatment with bz-ATP than GM astrocytes **(A)**. Primary rat microglia significantly increased their proliferation rate upon treatment with CCL2. This effect was abolished when the cells were co-incubated with a CCR2 antagonist **(B)**. Immunofluorescent double labelling study showed colocalization between BM28 and CCR2 in *post mortem* human hippocampal GML **(C)**. * p < 0,05.

The observed outnumbering of CCL2 positive cells in active WML compared to active GML was verified by an *in vitro* study. Upon stimulation with a disease-relevant stimulus, i.e. bz-ATP, primary rat WM astrocytes, showed a significant 6-fold increase in CCL2 mRNA compared to vehicle-treated (medium) astrocytes (*p* = 0.034). In contrast, GM-derived astrocytes showed a minimal increase in CCL2 mRNA upon bz-ATP stimulation (Figure 6A).

#### CCR2 mediated proliferation of GM-derived microglia

The relative absence of CCL2 production by GM astrocytes *in vivo* and *in vitro* urged the question whether the clearly present CCR2 in GM microglia is bound and activated by CCL2 and results in a functional response. Therefore, primary cortical rat microglia were treated with CCL2 in the absence or presence of the selective CCR2 antagonist INCB3344. Incubation of the microglia with INCB3344 only did not affect microglial proliferation. Upon CCL2 treatment, the proliferation of microglia was significantly increased (LSD, *p* = 0.04). In the presence of INCB3344, this CCL2-mediated increase in microglial proliferation was eliminated (Figure 6B). In support of a role for CCR2 in cell proliferation in GM of MS patients, we observed BM28 positive/CCR2 positive microglial cells in hippocampal GML (Figure 6C).

## Discussion

The present study demonstrates that CCL2 in astrocytes and its receptor CCR2 in monocytes/macrophages and microglia are significantly upregulated at the mRNA and protein level in hippocampal lesions of MS patients. Moreover, we show for the first time that there is a spatial and quantitative discrepancy in the distribution of CCL2 and CCR2 in the hippocampus. CCL2 and CCR2 appear in hippocampal WML whereas in GML only CCR2 is present, and more CCL2 and CCR2 positive cells are present in hippocampal WM compared to hippocampal GM. This discrepancy can contribute to the pathological differences observed in WML versus GML of MS patients and may possibly affect subsequent disease outcome.

Accumulating evidence shows that within the CNS of MS patients besides WML also GML, e.g. hippocampal lesions [8,9], are present which may explain functional deficits seen in MS patients [51,52]. Moreover, GML correlate better with various clinical parameters than WML [53-55]. It is therefore of utmost interest to understand the pathological features of GML versus WML, because that may give direction to the identification of novel therapeutic targets. An important pathological feature of WML is the infiltration of immune cells through the blood-brain barrier (BBB) into the CNS, which is considered to play a crucial role in the pathophysiology of MS [15-17]. In contrast, immunohistochemical studies indicate that the GM is not, or far less, affected by infiltrating immune cells [20]. Therefore, we hypothesized that an important mediator of immune cell infiltration, CCL2, and its receptor CCR2 are present in WML but to a lesser extent in affected GM regions in the hippocampus of MS patients.

Our observation that astrocytes are the source of CCL2 is in agreement with previous studies showing CCL2 expression by cells with an astrocytic morphology [36,56]. In line with the observation that CCL2 is more abundantly present in WM/WML is that WM-derived astrocytes produce significantly more CCL2 upon stimulation with bz*-*ATP *in vitro,* whereas GM-derived astrocytes hardly respond. This is not due to a total irresponsiveness of GM-derived astrocytes, since stimulation with pro-inflammatory cytokines interleukin-1β and tumor necrosis factor-α resulted in a strong increase in CCL2 mRNA expression (data not shown). Moreover, the low responsiveness to bz-ATP cannot be attributed to an absence of the P2X7 receptor for ATP on GM astrocytes, since we observed P2X7 receptor immunoreactivity in and on WM- and GM-derived astrocytes (data not shown). Still, it cannot be excluded that the observed difference in bz-ATP-mediated CCL2 production by WM- versus GM-derived astrocytes is due to variation in expression of purinergic receptors other than P2X7, though thus far no such heterogeneity between WM and GM astrocytes has been described [57]. The observed discrepancy in the number of CCL2 positive cells in hippocampal WM/WML versus GM/GML is in line with the observation that CCL2 mRNA levels are significantly higher within the corpus callosum (WM) compared to the cortex (GM) in both control mice as well as a mouse model for MS, e.g. cuprizone treated mice [58]. These differences in CCL2 expression by WM versus GM astrocytes might be explained by regionally dependent differences in astrocytes. Indeed, two different types of astrocytes have been recognized, i.e. fibrous astrocytes that reside mainly in the WM and protoplasmic astrocytes that are preferentially located in the GM [59]. The heterogeneity between fibrous and protoplasmic astrocytes is not restricted to morphology. Molecular differences between fibrous and protoplasmic astrocytes have been described as well (reviewed in [60]) with WM-derived astrocytes expressing more e.g. GFAP and glutamate transporter-1 (GLT-1) [61]. Such differences between astrocytes in WML versus GML as observed in the present study by heterogeneity in CCL2 expression and production can contribute to the variety in pathological and functional outcome of WML and GML.

An alternative explanation for the observed difference between CCL2 positive cell number in hippocampal WM vs hippocampal GM can be the local variation in presence of pro-inflammatory cytokines, e.g interleukin (IL)-1β. These cytokines increase CCL2 production by astrocytes *in vitro* [62] and could thus, besides ATP, contribute to the CCL2 producing astrocytes found in WML. Indeed, in WML of MS patients, IL-1β positive cells are present [63], but have thus far never been studied in GML. In rats suffering from relapsing EAE, IL-1β appears in certain GM areas, but no IL-1β was observed in the hippocampus of these animals [64], and this absence of IL-1β could contribute to the lack of CCL2 in GML. Since neurons located within the GM are known to suppress the production of pro-inflammatory factors [65-67], this could also indirectly prevent CCL2 production. Thus, astrocyte-specific and environmental conditions can explain the relative lack of CCL2 in GML. Since astrocyte-derived CCL2 is a prominent factor involved in the attraction of leukocytes through the BBB in WML in MS [25,26], the relative absence of CCL2 in GML may subsequently explain the lack of infiltrating immune cells in this type of lesion. Additionally, CCL2 has been described to interact with endothelial cells [68] consequently disrupting the BBB. Thus, the absence of CCL2 in the hippocampal GML could explain a lack of BBB damage in GML [69].

The present immunohistochemical study showed that CCR2 is observed in hippocampal WML as well as GML in myeloid-like cells. Moreover, CCR2 was significantly more expressed in hippocampal WML than in GML. Interestingly, fluorescent double-labelling of CCR2 and Iba1 indicated that in hippocampal WML CCR2 is expressed mainly by infiltrating monocytes/macrophages and amoeboid microglia, while in the GML CCR2 is expressed mainly by ramified microglia, which is in line with studies showing CCR2 expression by microglia and macrophages *in vitro* and *ex vivo*, which, however, did not differentiate between their regional origin [70-72]. This suggests that during MS lesion formation, the source of CCR2 positive cells in WML are both resident microglia and infiltrating monocytes/macrophages, while in the GML CCR2 positive cells are mainly resident microglia. Like our data for CCL2, the discrepancy between the higher number of CCR2 positive cells in hippocampal WM versus lower number in hippocampal GM might be explained by differences that reside within WM and GM, e.g. the WM harbors more microglia [73], which are more prone to be pro-inflammatory [74,75].

Another interesting finding is that we observed that CCL2 and CCR2 positive cells are more numerous in the border of the lesion with ongoing demyelinating activity and less in the demyelinated center of the lesion, irrespective of WML or GML. Indeed, in WML it has already been shown that CCL2 and CCR2 are mainly present at the border [35,36], whereas it was unknown for GML. Moreover, the present study showed that CCL2 and CCR2 immunoreactivity in the hippocampus was significantly less when either WML or GML were inactive. This has only been reported for CCL2 in WML [56]. Its receptor CCR2 has been shown to be mostly expressed during active inflammation and demyelination in relapsing EAE [71]. These findings support the notion that CCL2 expression by astrocytes plays an important role during the active phase of disease, and that CCL2-CCR2 interaction in this way likely contributes to infiltration of immune cells occurring during active WML formation in MS.

Although CCL2 was hardly observed in GML, its receptor CCR2 was upregulated in both active WML and GML. We questioned whether the CCR2 receptor expressed by microglia in GM can act as a functional receptor. To this end we studied CCL2-mediated GM microglial proliferation *in vitro*. Indeed, CCL2 induced a significant increase in the percentage of proliferating GM microglial cells, which was prevented by co-incubation with the specific CCR2 antagonist INCB3344. This indicates that GM-derived microglia express a functional CCR2 receptor, and thus CCL2-CCR2 mediated effects can occur in GML, e.g. the induction of microglial proliferation [30]. This was supported by our observation that CCR2 is expressed by BM28 positive proliferating microglia. Their localization at the border of GML suggests that the proliferating microglial cells are either a consequence of or contribute to GML formation. A recent study indicated that proliferating parenchymal microglia are the main source of microgliosis after ischaemic stroke [76]. However, the role of microglial proliferation during demyelination remains to be established. Since only nanomolar concentrations of CCL2 are needed to activate CCR2 [77], it is possible that, although no evident appearance of CCL2 positive cells in the GM was observed, increased presence of the CCR2 receptor increases the chance of CCL2-CCR2 interaction, and thus minute amounts of CCL2 can result in CCR2 mediated responses e.g. microglia proliferation in GML. Alternatively, it is even more likely that CCL2 diffuses from neighbouring WML into the GML to exert its effect. We can, however, not exclude the possibility that other chemokines, i.e. CCL8 or CCL16, known to be ligands for CCR2, interact with the receptor to induce *in vivo* effects, e.g. cell migration or proliferation, but no expression of these chemokines in MS GML has been described.

Overall, we conclude that the difference in CCL2 and CCR2 expression in hippocampal WML versus GML of MS patients, at least partly, explains the pathological differences observed in WML and GML. As such CCL2-CCR2 interaction is likely to be involved in leukocyte migration into the CNS contributing to WML formation, whereas the relative absence of CCL2 in GML of MS patients may explain the lack of infiltrating immune cells in this type of lesion. Based on our *in vitro* experiments, we propose that the CCR2 receptor in GM is a functional receptor and is involved in resident microglial cell proliferation, thereby contributing to microgliosis. Furthermore, the discrepancy in CCL2 and CCR2 expression in hippocampal WML and GML implies that the pathogenesis of WML formation differs from that of GML. As such, our data support several lines of evidence which already pointed towards a possible difference in the pathways underlying demyelination in WM and GM, as there is no, or only low, correlation between the extent of demyelination in WM and GM [6,11]. Although under debate, WML formation is considered to start with perivascular infiltration of CD4^+^ and CD8^+^ T-cells [16,78-81], whereas GML formation has been suggested to be initiated by meningeal inflammation [82,83]. Although this may hold true for GM cortical lesions in MS, this does not necessarily explain the occurrence of subcortical GML, e.g. hippocampal lesions. With our observations on the discrepancy in CCL2 and CCR2 expression in WML and GML we have added an additional factor that may explain or contribute to the pathways underlying WML and GML formation in MS.
